# Case Report: Viral Pneumonia Could Prompt the Advancement of Immune-Mediated Liver Disease

**DOI:** 10.3389/fmed.2021.582620

**Published:** 2021-05-17

**Authors:** Qian Li, Jun Wang, Xueshi Zhou, Hongzhou Lu, Mengji Lu, Lihua Huang

**Affiliations:** ^1^Department of Hepatology, The Fifth People's Hospital of Wuxi, Jiangnan University, Wuxi, China; ^2^Department of Infectious Diseases, Shanghai Public Health Clinical Center, Shanghai, China; ^3^Institute of Virology, University Hospital of Essen, University of Duisburg-Essen, Essen, Germany; ^4^Center of Clinical Laboratory, The Fifth People's Hospital of Wuxi, Jiangnan University, Wuxi, China

**Keywords:** influenza A, COVID-19, SARS-CoV-2, autoimmune hepatitis, liver cirrhosis

## Abstract

**Background:** The impact of the influenza A (H1N1) and SARS-CoV-2 virus on the development of autoimmune hepatitis has not been described previously.

**Methods:** In this case series, we evaluated the dynamic changes in liver function of three patients with autoimmune hepatitis who presented with viral infection (two with the H1N1 and one with the SARS-CoV-2 virus) during the recent COVID-19 outbreak.

**Result:** Patient 1 was a 68-year-old woman with a history of hepatitis of unknown origin before being infected with the H1N1 virus. Autoimmune hepatitis with an exacerbation of liver injury was diagnosed during the infection. Patient 2 was a 48-year-old woman with pre-existing autoimmune hepatitis. Despite being on immunosuppressant therapy (using glucocorticoids), liver injury recurred with elevated total bilirubin and gamma-glutamyl transferase levels post H1N1 infection. Patient 3 was a 61-year-old woman with probable autoimmune hepatitis. Liver injury recurred with a flare in alanine transaminase/aspartate transaminase levels post SARS-CoV-2 infection, in spite of the patient being on liver protection therapy (using ursodeoxycholic acid).

**Conclusion:** The case series raises the possibility that COVID-19 or influenza induced pneumonia triggers the progression of autoimmune hepatitis.

## Introduction

The outbreak of the coronavirus disease (COVID-19), which is caused by the highly contagious severe acute respiratory syndrome coronavirus 2 (SARS-CoV-2) infection, has become a global health threat. As of April 20, 2021, the COVID-19 has led to more than 140 million confirmed cases and 3 billion deaths worldwide (1). More importantly, another respiratory infectious disease known as influenza, caused by the influenza A virus, is known to share its etiology with COVID-19 and occurs in the same season (2). The pandemic of influenza A (H1N1) caused 151,700–575,400 deaths worldwide from 2009 to 2010. As a seasonal flu virus, it caused at least 34 million illnesses and 20,000 deaths from September 2019 to February 2020 (3, 4).

SARS-CoV-2 is an enveloped, positive-sense, single-stranded RNA beta-coronavirus with a 26–32 kb genome (5). The principal structural proteins of SARS-CoV-2 include spike (S) protein, membrane (M) protein, envelope (E) protein, and nucleocapsid (N) protein. The densely glycosylated S protein can bind with angiotensin-converting enzyme 2 (ACE2) as the host cell entry receptor. In addition to ACE2, the surface heparan sulfate (HS) is a crucial co-factor for ACE2-mediated SARS-CoV-2 entry (6, 7). In contrast, Influenza A viruses that infect humans is an enveloped, negative-sense, single-stranded RNA viruses with a around 13.5 kb genome (8, 9). The influenza A virus genome encodes about 10 proteins, which mainly include hemagglutinin (HA) and neuraminidase (NA) (10, 11). The HA and NA glycoproteins are essential for the receptor binding and viral fitness (12).

Both the two virus are major pathogens that primarily infect the human respiratory system, and leading to the respiratory symptoms from the mild respiratory illness to respiratory failure (13). Furthermore, patients with a severe case of COVID-19 and influenza A are at a high risk of acute respiratory distress syndrome (ARDS), multi-organ dysfunction, and mortality (14, 15). When clinicians compared the liver function of hospitalized patients with ARDS caused by COVID-19 with that caused by influenza (H1N1), liver dysfunction was reported in 45.2% of COVID-19 and 45.3% of H1N1 cases, which raises the possibility of significant viral pneumonia-induced liver injury (16). A more complicated clinical challenge, however, is the handling of patients with COVID-19 or influenza with pre-existing liver diseases, such as autoimmune hepatitis (AIH), due to their poor immune response and outcomes (17, 18). Since the impact of viral pneumonia on the progression of AIH is unclear, we herein report three cases of patients with pre-existing AIH who presented with a SARS-CoV-2 or H1N1 viral infection, for a better insight of this clinical scenario.

## Materials and Methods

The two cases of H1N1 viral infection were confirmed based on screening of a respiratory sample (a simple nose and/or throat swab) using the conventional and real-time quantitative RT-PCR based on the WHO's guidelines (19, 20). The COVID-19 case was confirmed based on positive detection of the SARS-CoV-2 in any one of the following: (1) high-throughput screening of nasopharyngeal swab specimens, (2) RT-PCR assay of the nasopharyngeal swab specimens, or (3) combined IgM-IgG assay of serum samples (21, 22). AIH is a form of immune-mediated liver disease, which is characterized based on the International Autoimmune Hepatitis Group criteria by elevated transaminase levels and hyperglobulinemia, with or without circulating autoantibodies (23, 24).

## Results

### Demographic and Clinical Characteristics of the Three Patients

#### Patient 1

A 68-year-old woman diagnosed with hepatitis of unknown origin after presenting with fatigue and anorexia previously was treated using liver protection drugs. After treatment, normal liver function was restored until she was infected with the H1N1 virus. She first presented with early symptoms of the infection (i.e., fever and cough) on 22 January 2020 (Table 1) and was positively diagnosed with influenza on 24 January 2020. Her chest computed tomography (CT) scan showed bilateral ground-glass opacities and consolidations, which are the common CT scan findings associated with influenza A induced pneumonia. Simultaneously, her symptoms of fatigue and anorexia reappeared. Laboratory results on admission showed lymphopenia, neutropenia, and elevated C-reactive protein (CRP) levels. Further, her alanine aminotransferase (ALT), aspartate transaminase (AST), and gamma-glutamyl transferase (GGT) levels were evidently elevated (Table 2).

**Table 1:** Onset of clinical manifestations and outcomes of patients admitted to hospital.

**Characteristics**	**Patient 1**	**Patient 2**	**Patient 3**
Age (year)	68	48	61
Gender	Female	Female	Female
Onset to admission(days)	3	2	7
**Initial symptoms**			
Fever	Yes	Yes	Yes
Cough	No	Yes	Yes
Sputum production	No	No	No
Dyspnea	No	No	Yes
Chest tightness	Yes	No	Yes
Hemoptysis	No	No	No
Headache	No	No	No
Chill or shivering	No	No	No
Myalgia	No	No	No
Fatigue	Yes	Yes	No
Nausea, anorexia	Yes	Yes	Yes
**Complications**			
Acute kidney injury	No	No	No
Liver dysfunction	Yes	Yes	Yes
Septic Shock	No	No	No
ARDS	No	No	Yes
Co-infection	*Candida albicans*	No	*Stenotrophomonas maltophilia*
**Treatment**			
ICU admission	Yes	Yes	Yes
Antiviral treatment	Yes	Yes	Yes
Antibiotic treatment	Yes	Yes	Yes
Glucocorticoids	No	Yes	Yes
Oxygen therapy	Yes	Yes	Yes
Non-invasive/invasive ventilation	No	No	Yes
**Outcomes**			
Days of hospitalization	11	25	39
**Organ failure assessment before infection**			
CLIF-OF Score	6	6	6
CLIF-SOFA Score	2	3	1
Apache II Score	7	8	8
**Organ failure assessment during infection**			
CLIF-OF Score	6	6	8
CLIF-SOFA Score	3	4	3
Apache II Score	13	12	13
**Organ failure assessment after treatment**			
CLIF-OF Score	6	6	6
CLIF-SOFA Score	3	2	2
Apache II Score	11	7	8

**Table 2:** Laboratory parameters of 3 cases hospital onset.

		**Patients 1**	**Patients 2**	**Patients 3**
**LABORATORY CHARACTERISTICS**				
**Blood routine**				
White blood cell (× 10^9^/L)	3.5-9.5	3.47	6.14	14.88
Neutrophil (× 10^9^/L)	1.8-6.3	0.783	0.77	3.75
Lymphocyte (× 10^9^/L)	1.1-3.2	0.46	0.101	1.23
Monocyte (× 10^9^/L)	0.1-0.6	0.28	0.75	0.57
C-reactive Protein (mg/L)	0.0-10.0	34.7	50.9	19.97
**Biochemical indicators**				
ALT (U/L)	4.0–44.0	201.3	34	142
AST (U/L)	8.0–38.0	169	31	49
Total Bilirubin (μmol/L)	2.0–21.0	17.5	29	17
ALP (U/L)	39–117	115	77	114
GGT (U/L)	5–40	180.3	64	200
Direct Bilirubin (μmol/L)	0–7.0	6.5	11.2	7.7
Serum Total Protein (g/L)	67.0–83.0	65	78.9	62
Serum Albumin (g/L)	35.0–50.0	41.8	44.9	33
Serum Creatinine (μmol/L)	35–115	52	49	31
**Blood coagulation function**				
D-dimer (mg/L)	0.0–0.5	0.33	0.49	1.22
PT (s)	11.5–15.5	13.4	13.3	13.6
APTT (s)	26.0–40.0	35.6	43	46.8
Fibrinogen (g/L)	2.0–4.0	2.88	2.88	6.16
TT (s)	14.0–21.0	17.8	18.8	16.5
**Blood gas analysis**				
PaCO_2_(mm Hg)	35.0–48.0	27	39	44
PaO_2_(mm Hg)	83.0–108.0	64	65	65
PaO2 / FiO2(mm Hg)	400.0–500.0	290.47	309.52	162.5

#### Patient 2

**Figure 1:**
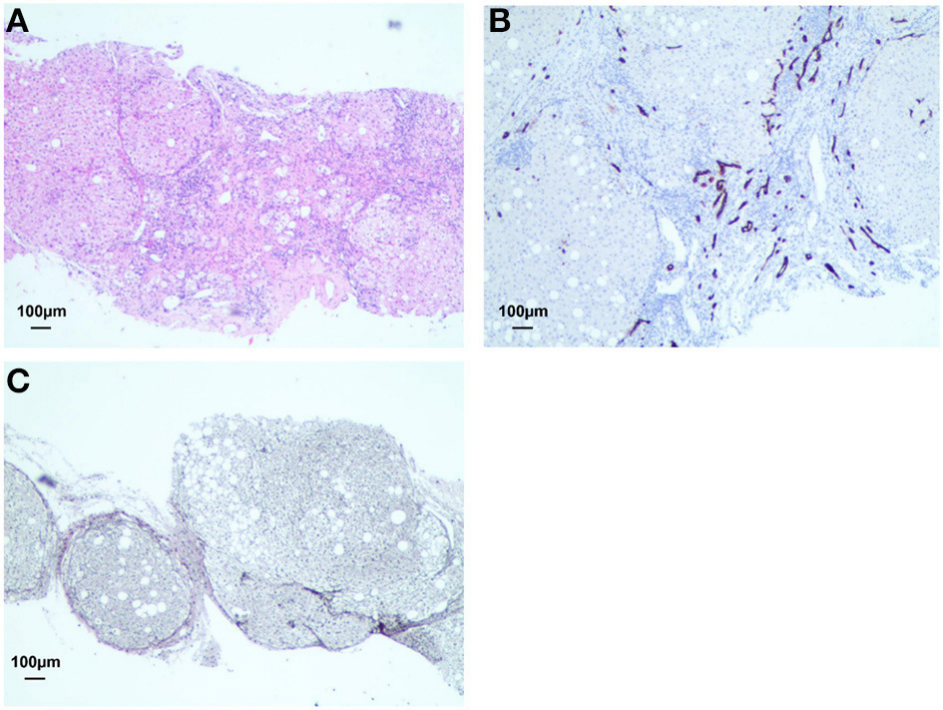
Lymphocytic infiltration with lymphoid follicles was accumulated and enlarged in the portal tract **(A)**. Cirrhosis was observed by immunolabeling with CD34 staining (brown) and Warthin-Starry (black) staining respectively **(B, C)**.

A 48-year-old woman was diagnosed with AIH (Child-Pugh class A; MELD score, 18) when she presented with the typical histological feature of interface hepatitis on Aug, 2018 (Figure 1). She was being treated for liver injury using methylprednisolone until she first presented with early symptoms of influenza (i.e., fever, cough, and dizziness) on 18 January 2020, and was positively diagnosed with influenza on 19 January 2020 (Table 1). Her chest CT scan showed similar bilateral ground-glass opacities and consolidations as that of patient 1. Moreover, she presented with fatigue, nausea, and anorexia, like patient 1, and further physical examination revealed liver palms, splenomegaly, and the feeling of an irregular liver on touch. Just as in the case of patient 1, patient 2's laboratory results on admission also showed lymphopenia, neutropenia, mononucleosis, and a high CRP level. Although the levels of some of her liver function parameters such as ALT, AST, and alkaline phosphatase (ALP) were in the normal range, the levels of others such as total bilirubin (TB), direct bilirubin, and GGT were elevated (Table 2).

#### Patient 3

A 61-year-old woman was suspected with AIH on September 2019 (Child-Pugh class A; MELD score, 12). On January 25, 2020, she presented with spiked fever that had persisted for seven days and she complained of tightness in her chest (Table 1). Owing to the persistent fever and ineffective antibiotic treatment, a chest CT scan was performed and revealed blurred margins in the left upper lung. A SARS-CoV-2-specific RT-PCR assay was performed, and a positive case of COVID-19 was confirmed on January 30, 2020. Her CT scan showed the hallmarks of a COVID-19 infection, namely bilateral ground-glass opacities. Further, her laboratory results on admission showed leukocytosis and elevated CRP, ALT, AST, GGT, and TB levels. In addition, her blood coagulation function parameters, including D-Dimer, activated partial thromboplastin time, and fibrinogen were elevated (Table 2).

### Liver Function Assessment During Viral Infection

**Figure 2:**
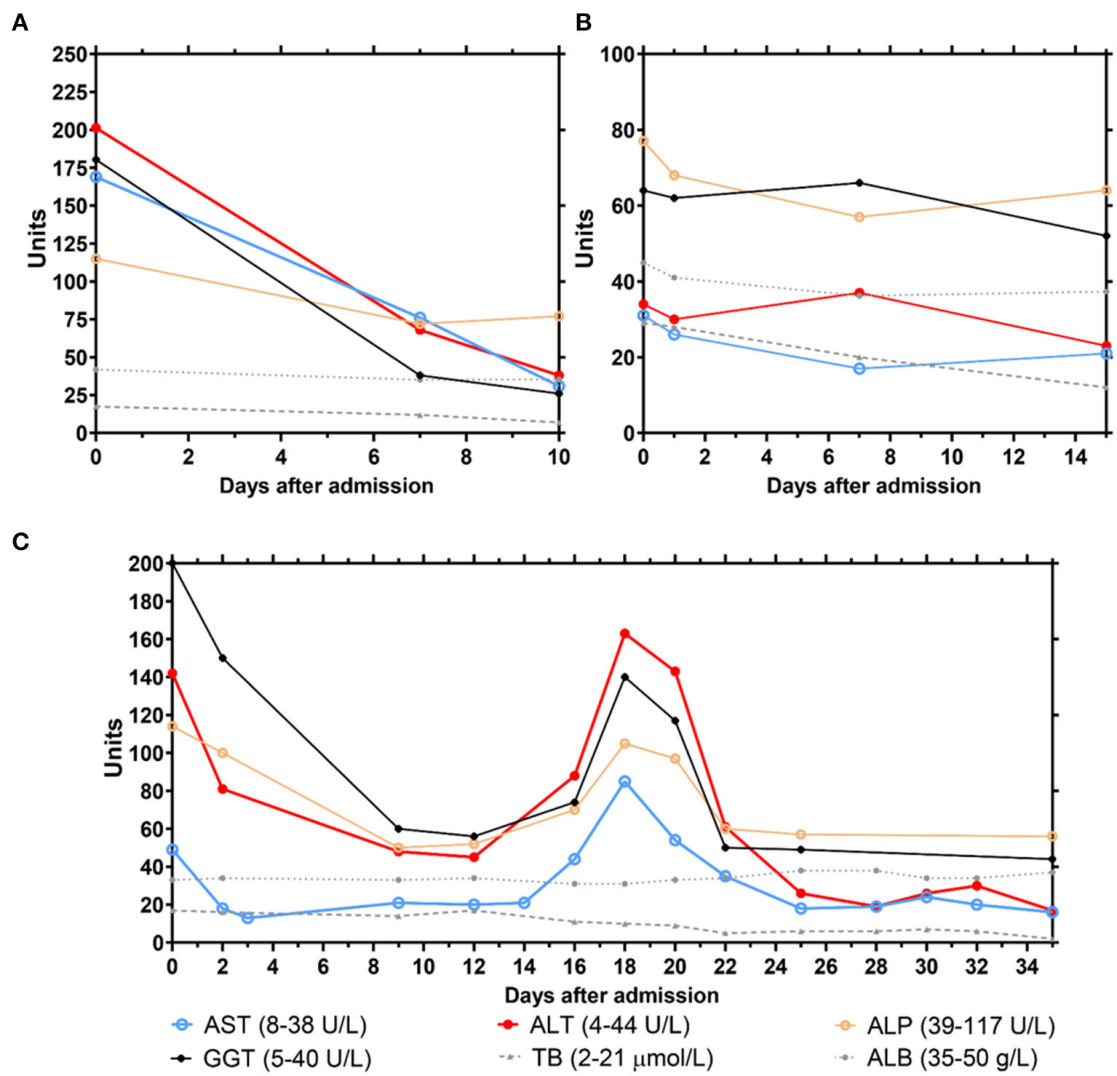
Monitoring of clinical parameters related to liver functions of the 3 cases (**A**, patient 1; **B**, patient 2; **C**, patient 3) during hospital treatment. Solid line, abnormal liver function parameters; dashed line, normal liver function parameters.

Patient 1 was evaluated with a low AIH score (AIH score: 8) with no antinuclear antibody (ANA), smooth muscle antibody, or liver/kidney microsomal type 1 (LKM-1) antibody detected. The baseline liver characteristics refer to her liver function assessment at hospital discharge, 3 months before she was diagnosed with influenza (Table 3). As is shown in Table 3, her MELD score was 7 and R Factor for liver injury was 1.3. After the onset of the H1N1 viral infection, her liver functions remarkably worsen. Initial laboratory tests revealed obviously elevated liver enzymes: AST, 169 U/L; ALT, 201.3 U/L; GGT, 180.3 U/L; and ALP, 115 U/L. Although viral serologies for hepatitis A, B, C, and E were still negative, serum immunoglobulin was 700.2 mg/dL (Figure 2A). Post H1N1 infection, her AIH score rose to 21 with a positive ANA titer of >1:80, and her liver histology revealed features typical of interface hepatitis. In addition, her MELD score and R Factor for liver injury increased to 13 and 5.3, respectively (Table 3).

**Table 3:** Liver function assessment related to viral infection.

**Characteristics**	**Patient 1**	**Patient 2**	**Patient 3**
**Liver assessment; scores before infection**			
AIH Score	8 (probable AIH)	18 (defined AIH)	12 (probable AIH)
Child–Pugh class	A	A	A
MELD score	7	7	10
R Factor for Liver Injury	1.3	1.3	1.0
**Liver assessment; scores during infection**			
AIH Score	21 (defined AIH)	19 (defined AIH)	14 (probable AIH)
Child–Pugh class	A	A	A
MELD score	13	13	12
R Factor for Liver Injury	5.3	1.6	3.7
**Liver assessment; scores after treatment**			
AIH Score	_	_	_
Child–Pugh class	A	A	A
MELD score	7	7	10
R Factor for Liver Injury	1.5	1.1	2

Patient 2 was diagnosed with defined AIH (AIH score: 18). Table 3 indicates her baseline liver characteristics, which refer to her liver function assessment at hospital discharge, 5 months before she was diagnosed with influenza. She was under glucocorticoid therapy until she was infected with the H1N1 virus. Due to this immunosuppressant therapy, although her ALT, AST, ALP, and total protein (TP) levels were in normal range, she had jaundice with elevated TB (29 μmol/L) and GGT (64 U/L) levels (Figure 2B). Her MELD score and R Factor for liver injury increased from 7 to 13 and from 1.3 to 1.6, respectively (Table 3).

Patient 3 was diagnosed with probable AIH (AIH score: 12). Table 3 indicates her baseline liver characteristics, which refer to her liver function assessment at hospital discharge, 3 months before she was diagnosed with COVID-19. Her MELD score and R Factor for liver injury were 10 and 1, respectively. From the onset of COVID-19 symptoms, her liver function deteriorated. Initial laboratory tests during COVID-19 hospitalization revealed evidently elevated liver enzymes: AST, 49 U/L; ALT, 142 U/L; GGT, 200 U/L; ALP, 114 U/L; and TP, 62 g/L (Figure 2C). Compared to her liver function before COVID-19, her AIH score elevated to 14 with a positive LKM-1 antibody titer>1:80. In addition, her MELD score and R Factor for liver injury increased to 12 and 3.7, respectively (Table 3). Once she was given systematic glucocorticoids normal liver function was restored. However, liver injury recurred with the highest level of ALT (163 U/L) and AST (85 U/L) after glucocorticoid withdrawal, and more liver protective drugs had to be administered.

### Therapy, Complications, and Prognosis

After infection, all patients received antiviral therapy: patient 1, Oseltamivir orally [75 mg, BID]; patient 2, first Oseltamivir orally [75 mg, BID] followed by pulsed Peramivir [0.6, ivgtt, qd] due to disease deterioration; and patient 3, ritonavir orally [400 mg/100 mg, BID] and interferon alfa-2b [500 wu, BID]. All patients were given systemic glucocorticoids combined with liver protective drugs (glutathione-based therapy) and broad-spectrum antibiotics. In addition, patients 1 and 2 were administered with oxygen inhalation due to their low PaO_2_ (<60 mmHg). Patient 3 required endotracheal intubation due to ARDS. During hospitalization, the sputum cultures of patient 1 and patient 3 showed the presence of *Candida albicans* and *Stenotrophomonas maltophilia*. Normal liver function was restored in all patients post therapy (Figure 2), and the liver assessment score decreased (Table 3). Finally, all patients were discharged from the hospital (patient 1, 1 February 2020; patient 2, 12 February 2020; and patient 3, 10 March 2020) after two consecutive negative results of the RT-PCR assays for H1N1 and SARS-CoV-2 were obtained on nasopharyngeal swabs (Table 1).

## Discussion

It has been reported in several studies that AIH may lead to cirrhosis and ultimately death due to an abnormal immune activation process (25). Understanding how H1N1 and SARS-CoV-2 viral infection has an impact on the development of AIH is important. This study mainly reported the liver function characteristics associated with viral pneumonia in patients with probable or pre-existing AIH. After the patients get infected with H1N1 or SARS-CoV-2, their score of liver function assessment (AIH score, MELD and R factor of liver injury) increases significantly, suggesting that viral pneumonia such as that caused by influenza and COVID-19, may potentially trigger the progress of AIH.

Patients with pre-existing liver disease such as AIH, presenting with COVID-19 or influenza, require proper treatment to prevent poor prognosis (16, 17). Even though different transmissibility and virulence of SARS-CoV2 and H1N1 owing to their construction and characters, usually lead to distinct clinical course during infection (26), all patients had an elevated liver function assessment score after viral pneumonia in this study. The observation suggests that a possible explanation for this may be the aberrant immune activation in the liver of patients with AIH (27). Thus, as pointed out by Lleo et al. (16) it is critical to pay special attention to any pre-existing liver diseases like AIH in patients with viral pneumonia.

The major limitation of our study is the small sample size. Therefore, we expect that further studies with a larger sample size will shed light on the clinical course of COVID-19 or influenza patients with pre-existing AIH. Therefore, we expect that further studies with a larger sample size will shed light on the clinical course of COVID-19 or influenza patients with pre-existing AIH. Besides, whether the viral infection induced liver injury in patients with pre-existing AIH is associated with the aberrant immune activation, and the exact mechanisms are unknown. Further experiments are needed to investigate the mechanisms of liver injury due to SARS-CoV2 and H1N1 infection with pre-existing AIH.

In conclusion, we report here the clinical course of patients with viral pneumonia associated with AIH. Our data suggests that viral pneumonia could prompt the progression of AIH.

## Data Availability Statement

The original contributions presented in the study are included in the article/supplementary material, further inquiries can be directed to the corresponding author/s.

## Ethics Statement

The studies involving human participants were reviewed and approved by The Ethics Committee of the Fifth People's Hospital. The patients/participants provided their written informed consent to participate in this study. Written informed consent was obtained from the individual(s) for the publication of any potentially identifiable images or data included in this article.

## Author Contributions

QL and JW: conceived and designed the study. ML, HL, and LH: drafted and revised the manuscript. XZ and LH: data collection. HL, ML and LH: data analysis and interpretation. All authors contributed to the article and approved the submitted version.

## Conflict of Interest

The authors declare that the research was conducted in the absence of any commercial or financial relationships that could be construed as a potential conflict of interest.
